# The Effect of Computerized Cognitive Training for Adults Over 40 with Dementia-Related Anxiety

**DOI:** 10.1093/geroni/igab046.3125

**Published:** 2021-12-17

**Authors:** Jennifer Roberts, Molly Maxfield

**Affiliations:** 1 University of Colorado Colorado Springs, Colorado Springs, Colorado, United States; 2 Arizona State University, Phoenix, Arizona, United States

## Abstract

Dementia-related anxiety (DRA) may occur when cognitive lapses are appraised as threatening. Individuals with DRA may seek activities to improve cognitive function, including popular computerized cognitive training programs like Lumosity©. We evaluated if DRA changed after eight weeks of Lumosity© use and whether changes were maintained over time. Participants aged 40 and older with pre-existing DRA participated via Amazon’s Mechanical Turk (T1 N = 395; age M = 52.49, SD = 8.71) and were randomly assigned to the experimental (Lumosity© software), active control (Lumosity© crossword puzzles), or no treatment group. Participants completed measures of DRA at T1 and at four follow-up points (T2 = 8 weeks; T3 = 12 weeks; T4 = 16 weeks; T5 = 20 weeks). Repeated measures ANOVAs were used to evaluate the change in DRA. A significant T1-T2 reduction in DRA occurred for the Lumosity© group only (p = .01, partial-eta2 = .03). Longitudinal changes were observed for the Lumosity© group only: DRA scores at T1 were significantly greater than at T2, T4, and T5 (ps < .05). A step-up test procedure was conducted to determine minimum treatment dose effects. A greater reduction in DRA occurred between the Lumosity© and crossword puzzle groups between 25.00 and 29.99 hours of software use (p = .05, partial-eta2 = .19). Lumosity© software outperformed crossword puzzles in DRA reduction from T1 to T2, which was maintained for 12 weeks post-software use. Independent of Lumosity’s intended purpose of supporting cognitive functioning, participants subjectively believe it helps and experience associated benefits.

